# Exploring the breadth of medicine: 8-year outcomes of a brief clinical summer immersion for premedical students

**DOI:** 10.1186/s12909-024-06301-5

**Published:** 2024-11-28

**Authors:** Eva Weinlander, Elizabeth Sams, Sarita Khemani, Armaan Jamal, Malathi Srinivasan

**Affiliations:** 1 Department of Medicine, Division of Primary Care and Population Health, Stanford University School of Medicine, Stanford Clinical Summer Internship, Stanford, CA USA; 2 Department of Medicine. COMET Fellowship, Division of Primary Care and Population Health, Stanford University School of Medicine, Stanford, CA USA; 3 Department of Medicine, Division of Hospital Medicine, Stanford University School of Medicine, Stanford, CA USA; 4 Division of Infectious Diseases, Johns Hopkins University School of Medicine, Baltimore, MD USA; 5 Department of Medicine, Center for Asian Health Research and Education, Stanford University School of Medicine, Stanford, CA USA

**Keywords:** Clinical Internship, Highschool students, College students, Premedical education, Premedical students, Pipeline Development, Healthcare Pipeline

## Abstract

**Background:**

Exposure to the breadth of healthcare opportunities is crucial to high-school and college students considering a career in medicine. Most programs revolve around research or subspecialties, limiting exposure to the richness within medicine.

**Objective:**

We conducted a program evaluation of the Stanford Clinical Summer Internship (CSI) 2-week program, to understand learner viewpoints around CSI program utility, and to assess long term impact. We assess viewpoints by learner level (high school versus college) and participation mode (in-person versus virtual).

**Methods:**

In 2016 we launched a two-week premedical internship, incorporating AAMC core competencies. In 2022 and 2023, we surveyed past participants, collecting demographic data and evaluating/comparing CSI’s impact on educational and career paths, future preferences in healthcare careers, and influential factors of matriculation for high-school and college participants.

**Results:**

Of 411 past participants, 42% responded (*n* = 173). We found minimal significant differences between high school and college students. The primary reason for joining was exploring a career in health professions. Notably, 82% acknowledged Stanford-CSI broadened their medical perspectives, 79% gained clarity on healthcare professionals' daily life, 79% heightened their interest in healthcare careers, 71% enhanced their resumes, and 72% learned valuable clinical skills. In-person participants reported developing more friendships (agree/strongly agree: 60% vs 35%, unpaired t-test: *p* = 0.01), while virtual participants reported having more interest in research careers (40% vs 68%, *p* = 0.01). Amongst high school matriculants (*n* = 133), 46% are now in college and 4% in medical or nursing school. Amongst collegiate matriculants (*n* = 40), 89% have graduated and 11% are now in graduate or medical school. All respondents believed Stanford-CSI was a worthwhile investment of time and resources, with nearly all reporting subsequent increased interest in medicine.

**Conclusions:**

Stanford-CSI's summer internship gives premedical students real-world medical profession exposure and fosters meaningful connections. Our findings and teaching framework can guide similar program developments, supporting future medical education initiatives.

**Supplementary Information:**

The online version contains supplementary material available at 10.1186/s12909-024-06301-5.

## Introduction

Medicine offers numerous rewarding career paths [1, 2]. Still, most premedical students encounter only a limited range of exposure to clinical practice during their early years, which may make it difficult for them to make an informed decision about pursuing medicine as a career [3–5]. Consequently, many premeds miss the opportunity to broaden their view on potential medical careers and to seek early experiences that hone their curiosity and skills for their future careers. To provide these early opportunities, many universities and health-systems have launched summer premedical immersion programs, introducing high school and college students to various healthcare careers. Many summer programs focus on specific subspecialties [2, 6, 7] or distinct research topics [3, 4, 7], spending one to four weeks training learners in a very specific area. Fewer summer programs provide a broader view of career opportunities within healthcare, by combining clinical and non-clinical medical exposure and role modeling.

In 2015, we launched the Stanford Clinical Summer Internship (Stanford-CSI) to provide early learners with a broad exposure to career possibilities within healthcare, including clinical skills training, direct observation and shadowing opportunities within several clinical specialties, hands on procedures and broad perspective workshops led by faculty in both clinical and non-clinical disciplines. We conducted program outreach through multiple channels, including the Stanford Medicine Summer Programs website, the American Academy of Medical College summer program website, the annual Stanford Science Fair, in-person student conferences, and other Stanford social media platforms. We also presented locally to high schools which focused on readying first-generation students from low-income families to attend and succeed in 4-year colleges.

We opened participation to premedical high school and college students, recognizing that both groups stand to gain equally from hands-on clinical experiences, foundational healthcare knowledge, and professional networking opportunities essential for pursuing a career in medicine [1, 8]. The goal of our program l was to ignite passion in young premedical students by offering them a wide-ranging look into the art, science, and joy of a medical career. In response to the COVID-19 pandemic, we transitioned to a fully virtual format in 2020 through 2022. From 2023 we offered one in person and one ZOOM [9] based program, which also allowed us to accommodate more students.

In this report, we share Stanford-CSI's eight-year outcomes, including participant feedback regarding their personal growth through program participation, and outcomes (where are they now). We also share our program's foundation, curriculum, lessons learned and key takeaways from both in-person and virtual implementations. In doing so, we hope that this program evaluation offers valuable insights and a teaching framework to support and guide the development of similar pre-med pipeline programs, fostering future medical education initiatives.

## Methods

### Setting

Stanford-CSI was developed within an academic health system with 4 affiliated hospitals (Stanford Hospital, Stanford Lucile Packard Children’s Hospital, Palo Alto Veterans Hospital and Stanford Healthcare TriValley Hospital), utilizing existing unique centers (e.g. Goodman Surgical Simulation Center, the Center for Advanced Pediatric and Perinatal Education, Stanford Cardiovascular Research Building Skills Lab, Stanford University Human Performance Lab), and the innovative teaching tools within Stanford’s Anatomy Lab and Stanford School of Medicine.

### Participant selection

Participants were selected from undergraduates and high-school juniors and seniors, using holistic review. Holistic review included academic standing, standardized test scores, two essays, extracurricular activities, and a professor or guidance counselor recommendation. Standardized test scores (SAT and ACT) were excluded from consideration during the pandemic and are optional for students to submit as of 2023. Classes were composed with attention to participant diversity (i.e. gender, geographic, learner level, ethnicity). Each class enrolled around 10% international students. Program fees vary yearly based on cost structure (virtual vs. in-person, facilities, faculty and staff time, lunches, and supplies). Need-based financial scholarships were initially awarded to 10% of non-international students, expanding to 14% in 2021 with the help of a matching division grant and donor support. By 2023, 20% of students received full program and/or residential aid. The availability and distribution of scholarships varied based on institutional and philanthropic funding. Once funding became available, need-based application waivers and scholarships were also granted to students requesting them who fell under the $80,000 poverty line and who contributed to creating a well-rounded and diverse class.

Current CSI program information and fees can be found at: https://med.stanford.edu/medcsi.html and https://med.stanford.edu/medcsi/about/cost.html.

### Program framework

**Fig. 1:**
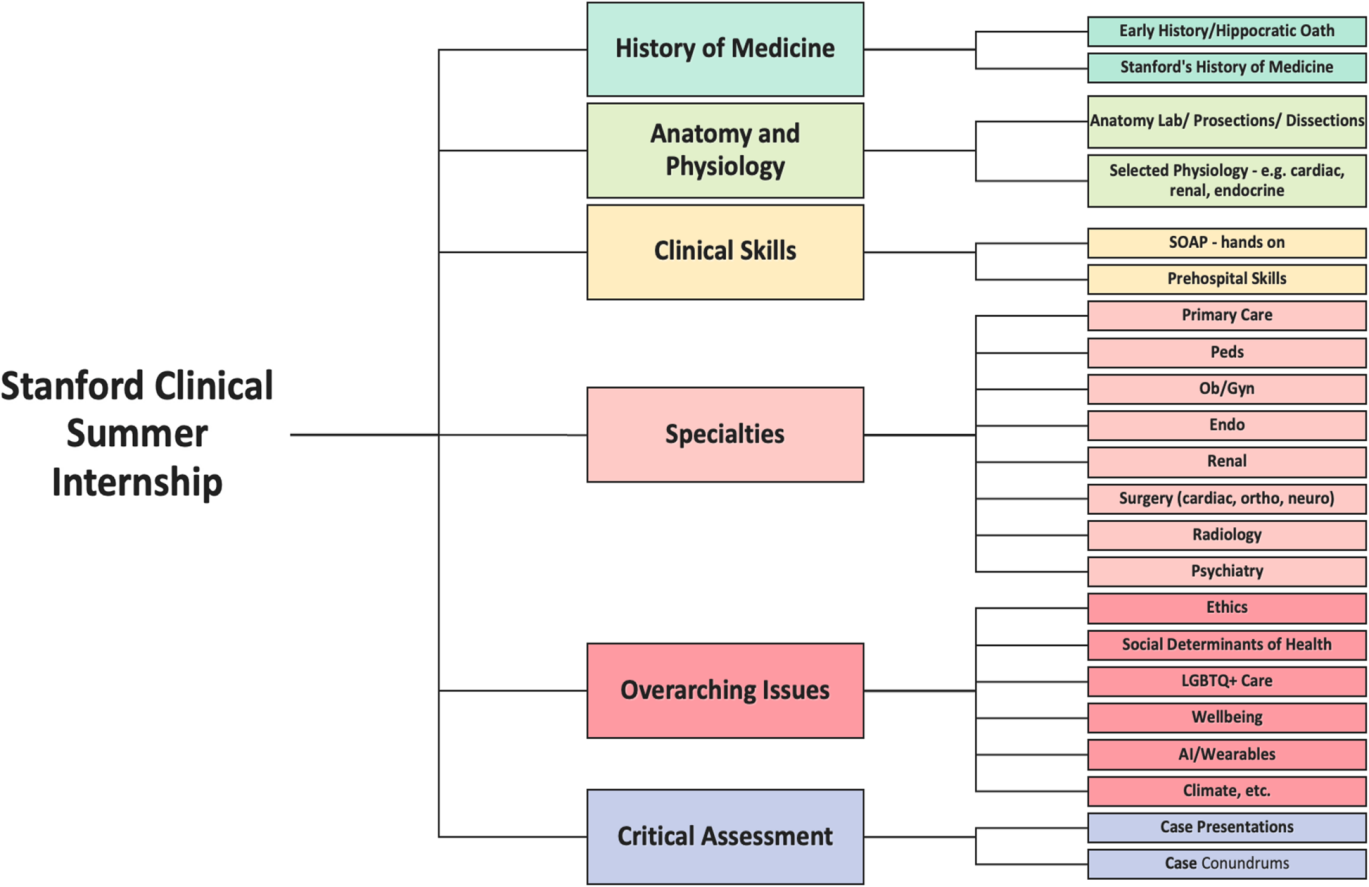
Stanford Clinical Summer Immersion (CSI) curricular map and schedule, based on American Academy Medical Colleges clinical competencies: Stanford-CSI Curricular map

Using the Association of American Medical Colleges (AAMC) core competency framework, we developed our core themes around critical thinking, teamwork, cultural competency, empathy, oral communication, scientific inquiry, and skills acquisition/exposure (Fig. 1). Within the two-week framework, our goal was to create structured, contextualized skills/roles exposure, stimulate learner curiosity and engagement, and help learners acquire appropriate and important first aid/prehospital skills.

### Program objectives

By the end of program participation, we hoped that learners would:

1. Understand multiple career options in medicine, including in primary care, specialty care, and healthcare research.
2. Develop a roadmap to successfully apply to medical or nurse practitioner/physician assistant school.
3. Establish relationships with Stanford faculty, medical and PA students, and program peers.
4. Be able to provide basic first aid and prehospital stabilization.
5. Gain experience in distilling and presenting complex medical information.
6. Be inspired to pursue a pathway in healthcare.

### Program overview

From 2015–2018, the Stanford-CSI was conducted in-person. A single session was offered in 2015, expanding to two the following years. Class size was kept small at 30 learners, to create an intimate learner-centered environment. Responding to participant feedback, a Stanford Campus residential component was added in 2017. In 2020, as a result of the pandemic, we pivoted to video-based Zoom format, expanding each of the two class sizes to 40, and then to 50 in 2021. Both in-person and remote students received a Stanford-CSI backpack containing a blood pressure cuff, stethoscope, tourniquet, and Stanford-CSI ware; Remote students also received preserved dissection specimens (animal heart, kidney, brain), suture/dissection kits, and a shopping list of optional material (ie. glucometer, oximeter). International students, unable to receive biological material, were sent a purchase list to enable full participation.

### Curricular structure

**Fig. 2:**
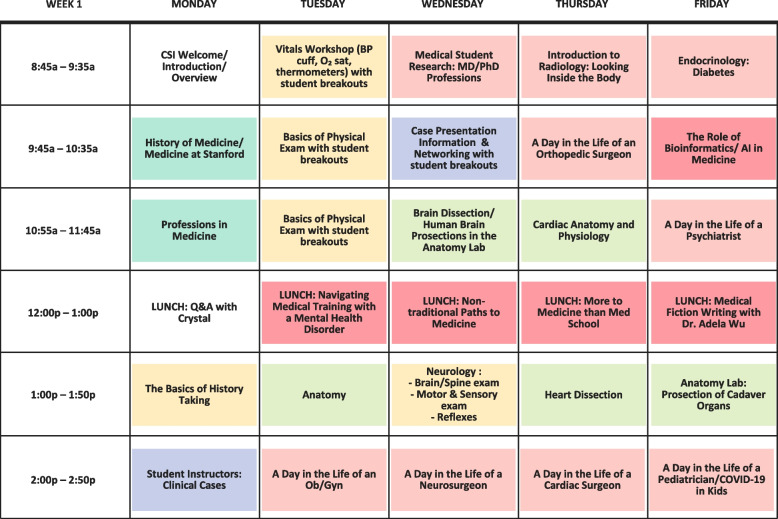
Stanford Clinical Summer Immersion (CSI) curricular map and schedule, based on American Academy Medical Colleges clinical competencies: Stanford-CSI curricular schedule week 1

**Fig. 3:**
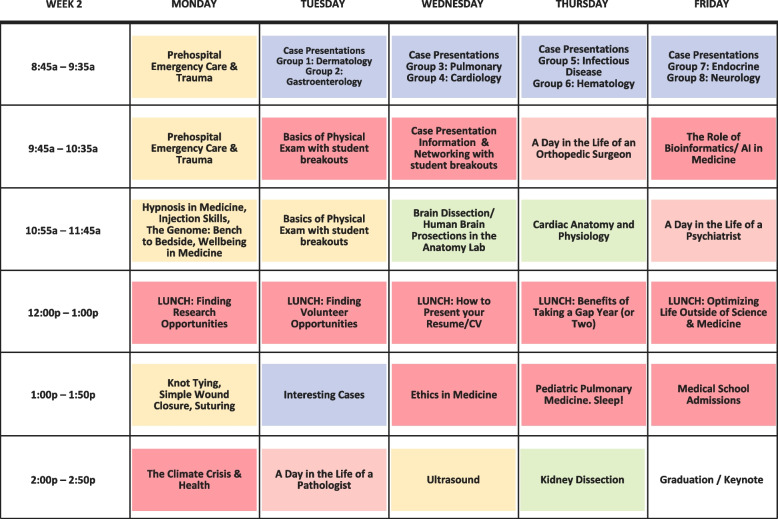
Stanford Clinical Summer Immersion (CSI) curricular map and schedule, based on American Academy Medical Colleges clinical competencies: Stanford-CSI curricular schedule week 2

We integrated core themes into all seminars, simulations, hands-on and skills-building workshops (Figs. 2 and 3). Clinical skills workshops were modeled after educational activities undertaken during medical school. In keeping with our clinical focus, participants practiced basic history and physical exam skills (with medical and physician assistant students, and faculty oversight). For dissections we provided pig hearts and kidneys, and sheep brains. For suturing we provided pigs feet and suture kits. We utilized existing resources including injection and venipuncture models, suturing kits, glucometers, bedside ultrasounds, obstetrics and neonatology simulations, prehospital emergency care supplies, virtual colonoscopy and minimally invasive surgical techniques in our surgical simulation lab. We often included current medical and physician assistant students to allow for mentorship opportunities, role-modeling and to decrease the learner to teacher ratio. In the Anatomy Lab, our students interacted with the Anatomage Table [10] and virtual reality headsets and were led through skeletal surveys and prosections by anatomy scholars. Since 2021 anatomy exposure has been restricted to virtual, but we plan to be allowed back in the lab in 2024. Participants practiced hands-on activities (e.g. vitals, physical exam skills and ultrasound) on each other, and during virtual sessions, were encouraged to do so with a friend, or family member.

We collaborated with experienced educators to showcase diverse healthcare career options and flexibility, in interactive medical lectures and “Day in the Life” seminars. We highlighted a broad array of healthcare specialties, including primary care, specialty medical and surgical care, psychology, psychiatry, nurse educators, dieticians, and pharmacists. Thought leaders in global health discussed social determinants of health and the intersection between health and climate.

When in person, student-teams had the opportunity to demonstrate the skills, knowledge and behaviors they had acquired in small team-based SOAP evaluations of a Standardized Patient (SP) with faculty oversight and SP feedback.

Students’ capstone projects were clinically focused. They worked individually to research specific medical diseases, including the S.O.A.P approach, distilling information down to how they might present during morning rounds, and sharing this summary prior to their system-based challenging case which was then navigated in small groups of 5–6, with a faculty facilitator, all in front of their peers.

Stanford faculty and staff available to students in-person for shadowing opportunities included: generalist & specialty physicians and primary care physician assistants and nurse practitioners. Physician specialties include physicians with expertise in family medicine, outpatient internal medicine, hospitalists, neurology, surgery (cardiac/plastics), urgent/express care, and critical care.

In lieu of the shadowing experiences for the virtual CSI program, Stanford faculty from multiple departments were invited to talk to the students about their professional life & experiences. We also had “office hours” with faculty, and PA/Medical students to share interesting cases.

### Program assessment

Post-course, at the end of each two-week session, we surveyed learners about course content, specific speaker feedback, learning climate, and their CSI experience, which was solely used for iterative course quality improvement. In 2022 & 2023, we re-surveyed all past participants about longer term program impact. The results from this alumni survey were analyzed for this program evaluation. Alumni were surveying anonymously through Qualtrics XM Survey Tool [11], with an 16 item survey that included multiple choice, Likert-like (five-point scale, strongly agree to strongly disagree), and open-ended questions (Supplemental Table 1). Students shared their reasons for program participation (two survey items), program contribution to perspectives regarding career opportunities and aspirations in health care (four survey items), use of their time and resources (three survey items), and program impact on educational and career path insights (four survey items). We also assessed the ability for virtual participants to engage with program material (two survey items). Participants were sent two follow up personalized email reminders.

Likert questions were collapsed to Agree (strongly agree, agree) vs Not Agree (neutral, disagree, strongly disagree), and presented as percent agreement. Responses are presented by level of education at time of matriculation as either high school or college/post-high school (gap year, college/university, post-graduate, working). Responses of in-person (2016–2019) and virtual participants (2020–2021) were compared in order to understand the impact of the mode of participation. To compare the response groups, Likert scale questions were coded on a scale from 1 to 5 (1 = strongly disagree to 5 = strongly agree). An unpaired T-test was then conducted to statistically analyze the significance of differences in responses between the two comparison groups, and the *p*-value was determined. Open-ended comments were analyzed using a rapid qualitative analysis [12, 13], using the lens of the Consolidated Framework for Implementation Research adapted for medical education, considering the inner and outer program settings, plus individual and intervention characteristics to understand program effects, strengths and areas for improvement [14, 15]. Two investigators (EW, LS) reviewed learner comments, distilled themes based on this construct, and resolved discrepancies through iterative discussion with the full investigator team.

This study was considered a program evaluation by the Stanford Institutional Review Board (IRB-76189). The program evaluation was conducted anonymously and completed voluntarily by prior program participants, and did not require specific consent to participate.

## Results

### Demographics

Of 411 prior Stanford-CSI participants, 220 (54%) responded after two prompts, with 172 (42%) completing all questions. Respondents included 77% high school students and 23% of college students. Among all respondents, the most recent CSI graduates had the highest response rate. Respondents identified as majority Caucasian (35%), Chinese (22%), Asian Indian (18%), or Hispanic/LatinX (11%). Respondents self-identified gender as 66% women, 31% male, 2.4% non-binary/other, without difference by learner level (Supplemental Table 2). We found no meaningful differences in responses by gender or race/ethnicity.

**Table 1:** Stanford Clinical Summer Internship: goals of matriculated students (2016—2023)

Student Goals for Stanford-CSI participation “Why did you attend the Stanford CSI program? (select your top 2 reasons)”	Total *n* = 172 n (%)	High School Participants *n* = 132 n (%)	College/ Post-High School Participants *n* = 40 n (%)
To consider a career in the health professions (medicine, nursing, PA, pharmacy, etc.)	143 (83%)	114 (86%)	29 (73%)
To consider a career in healthcare research	31 (18%)	28 21%	3.0 7.5%
To make connections with likeminded students	26 (15%)	20 (15%)	6.0 (15%)
To make connections with Stanford faculty as potential mentors	35 (20%)	24 (18%)	11 (28%)
To strengthen my resume	46 (27%)	32 (24%)	14 (35%)
To learn about specific specialties	46 (27%)	31 (24%)	15 (38%)
To learn about advances in science	10 (5.8%)	9.0 (6.8%)	1.0 (2.5%)
Other:	3.0 (1.7%)	1.0 (0.8%)	2.0 (5.0%)

### Matriculants

Most students joined the program to consider a career in health professions (83%), regardless of learner level (Table 1) . College students also reported participating to learn about specific specialties (38%), while high school students reported also participating as means to strengthen their resume (24%) in addition to learning about specific specialties (24%) (Table 2). Comparing responses from in-person participants from 2016–2019 to those from 2020–2021 who engaged through the entirely virtual format of Stanford-CSI (Table 3), in-person participants reported forming more friendships (agree/strongly agree: 60% vs 35%, unpaired t-test: *p* = 0.01). In contrast, virtual participants showed a higher interest in pursuing research careers (40% vs 68%, *p* = 0.01). Students participating virtually reported that it saved them time (63%), with half connecting meaningfully with instructors or faculty (51%) and just over one-third connecting meaningfully with classmates (35%) (Table 3). Amongst collegiate matriculants (*n*=40), 89% have graduated and 11% are in graduate or medical school as of the time of the survey. Of high school students, 46% have graduated, 46% are now in college, 4% students are currently enrolled in either medical or nursing school.

**Table 2:** Stanford Clinical Summer Internship outcomes: effect on learner perspectives and professional development (2016—2023)

Perspective Gained by Learner Level “Participation in the Stanford CSI program…*(% agree* = *agree* + *strongly agree)*	Total *n* = 173 n (%)	High School Participants *n* = 133 n(%)	College/ Post-High School *n* = 40 n(%)	Unpaired T-test
Broadened my perspective about healthcare	142 (82%)	108 (81%)	34 (85%)	< 0.1
Contributed to an increased interest in a career in healthcare	136 (79%)	100 (75%)	36 (90%)	0.4
Contributed to an increased interest in a career in specialty medicine	118 (68%)	86 (65%)	32 (80%)	0.5
Contributed to an increased interest in a career in primary care	84 (49%)	64 (48%)	20 (50%)	0.7
Contributed to an increased interest in a career in research	79 (46%)	62 (47%)	17 (43%)	0.5
Clarified what a life/career in medicine would be like	137 (79%)	102 (77%)	35 (88%)	0.8
Gave me valuable clinical skills	124 (72%)	93 (70%)	31 (78%)	0.8
Reinforced my career choice	120 (69%)	88 (66%)	32 (80%)	0.4
Changed my career choice	25 (14%)	22 (17%)	3 (8.0%)	< 0.1
Improved my professional network	74 (43%)	54 (41%)	20 (50%)	0.6
Improved my resume	123 (71%)	93 (70%)	30 (75%)	0.5
Led directly to new opportunities	60 (35%)	40 (30%)	20 (50%)	< 0.1
Led to new friendships	70 (40%)	55 (41%)	15 (38%)	0.3
Was a good use of my time	138 (80%)	102 (77%)	36 (90%)	0.4
Was a good use of my Resources	134 (92%)	98 (90%)	36 (97%)	0.2

**Table 3:** Stanford Clinical Summer Internship outcomes: In-Person Vs. Virtual Participant effect on learner perspectives and professional development (2016—2021)

Perspective Gained by Participation Mode “Participation in the Stanford CSI program… *(% agree* = *agree* + *strongly agree*	Total Participants *n* = 147 n(%)	In-person Participants *n* = 72 n(%)	Virtual Participants *n* = 75 n(%)	Unpaired T-test
Broadened my perspective about healthcare	140 (97%)	70 (97%)	70 (96%)	0.6
Contributed to an increased interest in a career in healthcare	134 (91%)	67 (93%)	67 (89%)	0.5
Contributed to an increased interest in a career in specialty medicine	117 (80%)	56 (78%)	61 (81%)	0.5
Contributed to an increased interest in a career in primary care	82 (57%)	35 (49%)	47 (64%)	0.1
Contributed to an increased interest in a career in research	78 (54%)	29 (40%)	49 (68%)	< .01
Clarified what a life/career in medicine would be like	135 (94%)	66 (92%)	69 (96%)	0.3
Gave me valuable clinical skills	122 (85%)	61 (85%)	61 (85%)	1.0
Reinforced my career choice	118 (82%)	55 (76%)	63 (88%)	0.1
Changed my career choice	25 (17%)	11 (15%)	14 (19%)	0.5
Improved my professional network	73 (51%)	35 (49%)	38 (53%)	0.6
Improved my resume	121 (84%)	60 (83%)	61 (85%)	0.8
Led directly to new opportunities	59 (41%)	27 (38%)	32 (45%)	0.4
Led to new friendships	68 (47%)	43 (60%)	25 (35%)	< .01
Was a good use of my time	136 (94%)	70 (97%)	66 (92%)	0.2
Was a good use of my resources	132 (92%)	68 (94%)	64 (89%)	0.2
“Participation in the Stanford CSI program in the virtual format….”				
Saved me time		N/A	50 (63%)	1.0
Let me connect with the instructors/faculty in a meaningful way		N/A	40 (51%)	1.0
Let me connect with my classmates in a meaningful way		N/A	28 (35%)	1.0

### Personal impact

Learners reported that Stanford-CSI broadened their perspective about healthcare (82%) (Table 3). College students reported that the program contributed to an increased interest in healthcare (90%), while high school students reported that the experience clarified what the daily life/career in medicine entailed (77%). Regardless of learner level, students reported that Stanford-CSI was a good use of their time and resources (80%). College students reported that their experience reinforced their career choice (80%), while high school students reported that the program gave them valuable clinical skills (70%). College students also indicated that their experience improved their professional network (50%) and led directly to new opportunities (50%). High school students reported that Stanford-CSI improved their professional network (41%) as well as led them to new friendships (41%).

Open-ended comments illustrated four major themes around program impact (Table 4):

- *Improved Career Perspectives:* Students reported that broadened perspectives enhanced their confidence in deciding to pursue a career in medicine.
- *Better Career Navigation:* Students gained valuable knowledge regarding necessary steps to pursue a variety of careers in medicine.
- *Expanded Professional Network*: Students expanded their professional networks through mentorships and connections with medical professionals.
- *Engagement in the Virtual Learning Environment:* Fostered meaningful engagement between students and faculty through online format.

**Table 4:** Stanford Clinical Summer Immersion participant viewpoints: representative quotes by theme and learner level at matriculation

Theme	Description	High School Participants	College Participants
Improved Career Perspectives	Students reported that broadened perspectives enhanced their confidence in deciding to pursue a career in medicine	“I never truly understood the broad range of professions in healthcare. Participating in this program prompted me to explore other career options. I was unsure of pursuing the medical field because of how long it would take me to be in a position where I can start helping others, but learning about paths that allow me to start working as early as possible reignited my passion to enter healthcare.”	“Stanford CSI participation helped deepen my understanding of the medical field and numerous specialties and their roles. The program reaffirmed my desire to become a physician. The program also taught me valuable clinician skills and strengthened my resume which led to other research internship opportunities.”
Better Career Navigation	Students gained valuable knowledge regarding necessary steps to pursue a variety of careers in medicine	“Stanford CSI provided me with a much deeper understanding of not only the field of medicine but also the path to becoming involved in that field and the various lifestyles it could lead to. It helped me clarify my choice of major and career in a more holistic way, considering the emotional and mental aspects of a career in medicine, alongside the academic ones. The program also motivated me to seek out more healthcare-related activities, such as observerships, by highlighting the importance of a hands-on, activity-based approach to understanding the medical field.”	“Stanford CSI gave me a much more in-depth understanding of my path towards medicine, but also in how I could tailor a career to fit my personal interests and passions. Shadowing at the Pacific Free Clinic gave me a taste of the sort of medicine I hoped to practice, and the time speaking with welcoming staff, residents, and med students left me curious and inspired. Aside from helping me confirm my interest in medicine, it gave me perspective on the incredible variety available within the career. The supportive environment became a cornerstone in my confidence moving toward medicine.”
Expanded Professional Network	Students expanded their professional networks through mentorship and connections with medical professionals	“Two weeks felt short but it was the perfect amount of time. I appreciated the time to meet and socialize with others in the program during lunch – I made lifelong friendships there! During the program, I met a mentor there who I still keep very closely in touch with. As a clinical researcher applying to MSTP programs	“Through Stanford faculty, I connected with other medical professionals and have been a part of wonderful research and shadowing opportunities.”
		this upcoming cycle, the power of networking in the clinical and medical fields have helped me substantially.”	
Engagement in the Virtual Learning Environment	Fostered meaningful engagement between students and faculty through online format	“I am so thankful that Stanford CSI exposed me to the scientific knowledge and occupational routines of so many different medical specialties. The internship strongly reinforced my excitement and wonder regarding the field of medicine. I am most grateful for the hands-on demonstrations, dissections, and techniques (such as suturing) that we practiced during the 2020 virtual internship. All of these were completely new to me and gave me first hand exposure to an entirely new aspect of medicine I had never encountered previously.”	“Participating in Stanford CSI was one of my best decisions yet. I learned so many things about careers in medicine and clinicals that have stuck with me thus far. Hearing about the experience being remote, I was worried about how interactive it would be. However, Stanford made the program the most insightful remote learning experience I have ever had, and that’s a lot given my last two years of schooling have been online! This program also motivates me to pursue an MD/PhD program and other areas of medicine. I learned so many things and met lots of people while being a part of this amazing program.”

## Discussion

Participants in our brief clinical immersion program gained a deeper understanding of the healthcare career pathway, while acquiring basic skills in medical history-taking, physical examination, and pre-hospital emergency care. In-person participation enhanced interpersonal communication and networking. Through shadowing experiences, participants gained an authentic glimpse into the day-to-day lives of healthcare professionals, highlighting clinical, teaching, and research and administrative opportunities and challenges. Participants reported a sustained interest in the health professions and affirmed the program’s enduring influence on their career outlook.

Early learners exploring healthcare careers can choose from a variety of high-quality summer immersion choices, with a range of durations, and reflecting the many roles healthcare professionals play improving health and public welfare (Table 4). For instance, the Wilderness and Emergency Medicine program at the University of Colorado School of Medicine offers undergraduates a chance to learn medical skills with a focus on wilderness medicine and global health, all within an intensive 1.5-week program [16]. The Georgetown University School of Medicine’s three-Week Medical Academy offers high school students a selection of three tracks as their chosen area of study (Anatomy & Physiology, Neuroscience, or Emergency Medicine) [17]. Similarly, Tufts University School of Medicine's Mini-Med School provides high school students aspiring to become physicians the chance to work with medical student mentors on engaging medical case studies and hands-on microbiology labs, while also offering insights into the MD admissions process over a brief two-week period [18]. Stanford-CSI’s two-week duration is designed to be accessible, allowing a wider range of students to participate without the need for a lengthy commitment [2]. This overview may spark interest that students can subsequently explore in depth.

For instance, 11th and 12th grade students participating in Boston University School of Medicine’s *AIM: Intro to Medicine* 4-week program reported forming valuable connections with students and faculty [19]. High school seniors and college freshmen participating in Drexel University College of Medicine's three-week Mini-Medical School program reported that clinical shadowing experiences were influential in their decision to pursue a career in medicine [20]. High school and college students participating in the Stanford-CSI program reported value in the clinical exposure and expanded professional network, for all learner levels.

Both in-person and virtual programs have had deep engagement from learners and teaching faculty. After rapidly transitioning to a ZOOM- based virtual teaching in 2020, we learned new methods to deeply engage students to help them effectively learn clinical skills and content. On-line and in-person, we Interspersed seminars with hands-on activities that promote learner engagement. We paid close attention to learner climate and drew quieter students into discussion in small-groups and through activities that promote collaborative learning. We implemented robust procedures for handling student illnesses for virtual and on campus courses. To ensure a seamless experience for participants, we now prepare backup lectures in advance to accommodate last-minute speaker cancellations. The virtual program, which we have continued post pandemic, allowed us to improve access to more students including those in geographically remote areas, at a lower cost, and save students from incurring costs for travel, room and board.

Our program evaluation has several limitations. By design, short-course programs provide exposure to, but not competency in, a given field. Recent CSI graduates showed the highest survey response rates, which gradually decreased over time, suggesting self-selection bias. Early Stanford-CSI participants may have difficulty recalling their initial feelings about the program (recall bias) but may be better positioned to describe program impact. We found survey drop-off towards the end of the survey, potentially impacting generalizability of our findings. While anonymous, respondents may be influenced by social desirability bias. Aimed at supporting learner’s interest in medicine, participation was likely motivated by an already established interest in medicine. Another limitation is the affordability of the program, which restricts its accessibility. While several need-based scholarships were offered annually, with the number increasing since the establishment of Stanford-CSI, program costs may still limit participation. We also recognize that the decision of participants to continue pursuing a career in medicine following Stanford-CSI only reflects one of the many influences shaping their professional aspirations.

## Conclusion

Early exposure to the broad array of healthcare opportunities, by dedicated and experienced faculty, positively influenced our learners’ career perspectives. By introducing a broad clinical exposure, our program may provide a model to attract a rich variety of cultural and societal perspectives for early learners interested in healthcare. For instance, we collaborated with ValleyCare Hospital, a Stanford affiliate, to create a similar CSI program, now in its third year. In Fall 2023, we added an afterschool virtual CSI Bootcamp. We are also exploring a year-round longitudinal format for local students to gain a deeper exposure to clinical medicine and meaningful shadowing experiences. We hope that early exposure to a broad array of healthcare opportunities, with dedicated and experienced faculty showcasing the art, science, and joy inherent in medical careers will inspire our young learners.

## Supplementary Information

Additional file 1: Supplemental Table 1. Stanford Clinical Summer Internship participant evaluation survey.

Additional file 2: Supplemental Table 2. Stanford Clinical Summer Internship participant demographics (survey respondents, 2016-2023).

Additional file 3: Supplemental Table 3. Characteristics of brief United States clinical programs for premedical students (1-3 week durations).

## Data Availability

No datasets were generated or analysed during the current study.
