# Supplementary material for: Exploring the breadth of medicine: 8-year outcomes of a brief clinical summer immersion for premedical students
Source: BMC Med Educ. 2024 Nov 28;24:1387. doi: 10.1186/s12909-024-06301-5 (PMC11606093; doi:10.1186/s12909-024-06301-5)
Supplement: Supplementary file 1 — Additional file 1: Supplemental Table 1. Stanford Clinical Summer Internship participant evaluation survey. [file 12909_2024_6301_MOESM1_ESM.pdf]

Stanford CSI Follow-up Learner Survey

Start of Block: Default Question Block

Intro Dear Stanford CSI Alumni,

We hope that you and your families are healthy and thriving in these challenging times.

Time has flown! Seven years ago, we started the Stanford Clinical Summer Internship, and have met the most amazing students – you! Now, we would like to understand what you are doing, and how Stanford-CSI was useful for you.

We would be very grateful if you could take 5 minutes to complete this anonymous survey. We will use your responses to revisit and improve our program.

Warmly,
Drs. Khemani and Weinlander and the Stanford-CSI team

Page Break

Q1 In what year did you participate in the Stanford CSI Program?

- ○ 2016 (coded as 1)
- ○ 2017 (2)
- ○ 2018 (3)
- ○ 2019 (4)
- ○ 2020 (5)
- ○ 2021 (6)

Display This Question:

If In what year did you participate in the Stanford CSI Program? = 2020

Or In what year did you participate in the Stanford CSI Program? = 2021

Q19 Stanford's CSI is usually held in person, and we would like to understand how the shift to virtual education affected your experience. How strongly do you agree or disagree with the following statements?

Participating in the Stanford CSI program virtually….

	Strongly Disagree (1)	Disagree (2)	Neutral (3)	Agree (4)	Strongly Agree (5)
Saved me time (coded as 1)	○	○	○	○	○
Let me connect with the instructors/faculty in a meaningful way (2)	○	○	○	○	○
Let me connect with my classmates in a meaningful way (3)	○	○	○	○	○

Q2 What academic year did you enter following your participation in Stanford CSI?

- ○ High School – Sophomore (coded as 1)
- ○ High School – Junior (2)
- ○ High School – Senior (3)
- ○ College – Freshman (4)
- ○ College – Sophomore (5)
- ○ College – Junior (6)
- ○ College- Senior (7)
- ○ Other (8) ____

Q3 With which gender do you identify? (select one)

- ○ Male (coded as 1)
- ○ Female (2)
- ○ Non-binary (3)
- ○ Other: (4) ____
- ○ Prefer not to say (5)

Q4 With which race/ethnicity do you identify? (Select all that apply)

- ☐ Hispanic/Latinx (coded as 1)
- ☐ Mexican (2)
- ☐ Puerto Rican (3)
- ☐ Caucasian or Non-Hispanic white (4)
- ☐ African American or Non-Hispanic black (5)
- ☐ Middle Eastern (6)
- ☐ Chinese (8)
- ☐ Asian Indian (9)
- ☐ Filipino (10)
- ☐ Vietnamese (11)
- ☐ Japanese (12)
- ☐ Korean (14)
- ☐ Native American (17)
- ☐ Native Hawaiian (20)
- ☐ Other Pacific Islander (16)____
- ☐ Other Asian (15) ____
- ☐ Other: (18) ____
- ☐ Prefer not to answer (19)

Q5 At the time of the program, did you request financial assistance?

- ○ Yes (coded as 1)
- ○ No (2)

---

*

Q6 Why did you attend the Stanford CSI program? (select your top 2)

- ☐ To consider a career in the health professions (medicine, nursing, PA, pharmacy, etc) (coded as 1)
- ☐ To consider a career in healthcare research (2)
- ☐ To make connections with like-minded students (3)
- ☐ To make connections with Stanford faculty as potential mentors (4)
- ☐ To strengthen my resume (5)
- ☐ To learn about specific specialties (6)
- ☐ To learn about advances in science (7)
- ☐ Other: (8) ____

Page Break

Q7 What are you doing now? (select all that apply)

- ○ High School (coded as 1)
- ○ College (2)
- ○ Gap year/time (3)
- ○ Working (4)
- ○ Master’s Program (5)
- ○ PhD Program (6)
- ○ Medical School (7)
- ○ Nursing School (8)
- ○ Nurse Practitioner/Physician Assistant School (9)
- ○ Other: (10) ____

Q8 How strongly do you disagree or agree with the following statements?
"Participation in CSI…

	Strongly Disagree (1)	Disagree (2)	Neutral (3)	Agree (4)	Strongly Agree (6)
Broadened my perspective about healthcare (coded as 1)	◯	◯	◯	◯	◯
Contributed to an increased interest in a career in healthcare (2)	◯	◯	◯	◯	◯
Contributed to an increased interest in a career in specialty medicine (3)	◯	◯	◯	◯	◯
Contributed to an increased interest in a career in primary care (4)	◯	◯	◯	◯	◯
Contributed to an increased interest in a career in research (5)	◯	◯	◯	◯	◯
Clarified what a life/career in medicine would be like (6)	◯	◯	◯	◯	◯
Gave me valuable clinical skills (7)	◯	◯	◯	◯	◯
Reinforced my career choice (8)	◯	◯	◯	◯	◯
Changed my career choice (9)	◯	◯	◯	◯	◯
Improved my professional network (10)	◯	◯	◯	◯	◯
Improved my resume (11)	◯	◯	◯	◯	◯
Led directly to new opportunities (12)	◯	◯	◯	◯	◯
Led to new friendships (13)	◯	◯	◯	◯	◯
Was a good use of my time (14)	◯	◯	◯	◯	◯
Was a good use of my resources (15)	◯	◯	◯	◯	◯

Q9 How did Stanford CSI participation impact your career insights and development, and your understanding of the field of medicine?

____

____

____

____

____

End of Block: Default Question Block

Start of Block: Final Thoughts

Q11 Any final thoughts or reflections about CSI? Experiences that you liked? Things to change?

____

____

____

____

____

End of Block: Final Thoughts
