# Supplementary material for: Exploring the breadth of medicine: 8-year outcomes of a brief clinical summer immersion for premedical students
Source: BMC Med Educ. 2024 Nov 28;24:1387. doi: 10.1186/s12909-024-06301-5 (PMC11606093; doi:10.1186/s12909-024-06301-5)
Supplement: Supplementary file 2 — Additional file 2: Supplemental Table 2. Stanford Clinical Summer Internship participant demographics (survey respondents, 2016-2023). [file 12909_2024_6301_MOESM2_ESM.pdf]

Supplemental Table 2. Stanford Clinical Summer Internship participant demographics (survey respondents, 2016-2023).

Expression	Total n=172 n(%)	High School Participants n=132 n(%)	College/ Post-High School Participants n=40 n(%)
**Self-Identified Gender**			
Female	114 (66%)	83 (63%)	31 (78%)
Male	54 (31%)	46 (35%)	8.0 (20%)
Non-binary	2.0 (1.2%)	1.0 (0.8%)	1.0 (2.5%)
Other/Prefer not to say	2.0 (1.2%)	2.0 (1.5%)	0.0 (0.0%)
**Self-Identified Race/Ethnicity**			
Caucasian/Non-Hispanic white	60 (35%)	45 (34%)	15 (37%)
Chinese	34 (22%)	26 (23%)	8.0 (21%)
Asian Indian	28 (18%)	23 (20%)	5.0 (13%)
Hispanic/Latinx*	18 (11%)	12 (9.1%)	6.0 (15%)
Middle Eastern	7.0 (4.1%)	4.0 (3.0%)	3.0 (7.5%)
Korean	10 (5.8%)	10 (7.6%)	0.0 (0.0%)
African American/ Non-Hispanic black	7.0 (4.1%)	3.0 (2.3%)	4.0 (10%)
Filipino	4.0 (2.3%)	4.0 (3.0%)	0.0 (0.0%)
Vietnamese	4.0 (2.3%)	2.0 (1.5%)	2.0 (5.0%)
Japanese	2.0 (1.2%)	2.0 (1.5%)	0.0 (0.0%)
Native American	1.0 (0.6%)	0.0 (0.0%)	1.0 (2.5%)
Other/Prefer not to say	11 (6.0%)	6.0 (5.0%)	5 (13%)

*Hispanic/Latinx includes Mexican and Puerto Rican.
