# Supplementary material for: Exploring the breadth of medicine: 8-year outcomes of a brief clinical summer immersion for premedical students
Source: BMC Med Educ. 2024 Nov 28;24:1387. doi: 10.1186/s12909-024-06301-5 (PMC11606093; doi:10.1186/s12909-024-06301-5)
Supplement: Supplementary file 3 — Additional file 3: Supplemental Table 3. Characteristics of brief United States clinical programs for premedical students (1-3 week durations). [file 12909_2024_6301_MOESM3_ESM.pdf]

Supplemental Table 3. Characteristics of brief United States clinical programs for premedical students (1-3 week durations).

Institution	Program	Duration	Learner Level	Program Focus	Featured Attributes	Website
University of Connecticut	Pre-Med: Explore the Medical Field	1 week	15+ yo	Focus on Overview of cardiology and gastroenterology. Insights into the daily life of a physician, to help students decide if medicine is a potential career path.	Pre-med academic advising. Professional Networking. Clinical skills training.	UCONN pre-med
Highland Hospital in Oakland, CA	Medical Immersion Summer Academy (MISA)	1 week	Rising 10th, 11th, 12th	Exposure to broad range of healthcare and clinical experiences	Clinical skills training. CPR certification. Professional Networking.	MISA
Tufts University School of Medicine	Mini-Med School	1 week	Rising 11th, 12th, Undergrads	Exposure to broad range of clinical experiences and career options in medicine	Clinical skills training. Anatomy lab	Tufts mini-med school 1 week

Institution	Program	Duration	Learner Level	Program Focus	Featured Attributes	Website
University of Colorado School of Medicine	Wilderness and Emergency Medicine	1.5 weeks	Undergrads, gap year students, and recent graduates	Emphasis on emergency/wilderness medicine and global health. Provide students with info and tools to help medical school applications.	EMT certificate<br>Clinical skills training (EMT)<br>Professional Networking.	Wilderness and Enviro Med
Stanford University School of Medicine	Stanford Anesthesia Summer Institute (SASI)	1 or 2 weeks	11th, 12th Undergrads (must be 16+)	Anesthesia, perioperative care,<br><br>Capstone	Optional virtual Capstone project.	SASI
Stanford University School of Medicine	Stanford Clinical Summer Internship (Stanford-CSI)	2 weeks	11th, 12th Undergrads (must be 16+)	Broad intro to medicine and career opportunities. Shadowing opportunities.	Lectures.<br>Clinical skills, Anatomy Lab.<br>Physician Shadowing.<br>Simulation (peds trauma/childbirth<br>Capstone project.<br>Professional networking.	Stanford CSI

**Institution**	**Program**	**Duration**	**Learner Level**	**Program Focus**	**Featured Attributes**	**Website**
Stanford University School of Medicine	Stanford Cardiothoracic Surgical Skills Summer Internship	2 weeks	16+ yo	Provide knowledge and exposure to basic cardiothoracic surgery and technical skills	Lectures.<br>Clinical skills training in CT surgery.<br>*Virtual only.*	Stanford CSS
Tufts University School of Medicine	Mini-Med School	2 weeks	Rising 11th, 12th + Undergrads	Experience in studying the medical sciences, focused on infectious disease and immunology.<br>Explore career options in medicine.	Lectures.<br>Anatomy lab .<br>Clinical skills training.<br>Capstone project.	Tufts mini-med school
University of California-Irvine	Summer Surgical Program	2 weeks	16+ yo	Hands-on experience in surgery and medicine	Surgical shadowing<br>CPR & BLS certification<br>Anatomy lab	UC Irvine HS Program

Institution	Program	Duration	Learner Level	Program Focus	Featured Attributes	Website
Boston University	AIM: Intro to Medicine	3 weeks	11th, 12th	Broad intro to medicine career opportunities. Science coursework and experiential activities	Simulation labs Clinical Skills training Anatomy lab	AIM BU
University of California	Program for Investigation and Training in Careers in Health (PITCH)	3 weeks	Rising 9th, 10th, 11th, 12th	Catered for students with underrepresented backgrounds. Emphasis on dentistry, pharmacology, medicine, and nursing	Grad student mentoring. Professional Networking. Lectures Capstone project.	UCSF PITCH
Georgetown University	Medical Academy	3 weeks	Rising 9th, 10th, 11th, 12th	3 tracks: anatomy & physiology, neuroscience, or emergency med	Case studies & labs. Capstone project.	Georgetown MA

Institution	Program	Duration	Learner Level	Program Focus	Featured Attributes	Website
University of Pittsburgh	Health Career Scholars Academy	4 weeks	Rising 11th, 12th	Offers health-relevant concentration courses for students to study in depth	Lecture courses offered vary by year (ie. health care for the chronically ill, family and child health, behavioral health, geriatrics, global health and public health)	HCSA
